# Supplementary figures and images for: A lytic transglycosylase connects bacterial focal adhesion complexes to the peptidoglycan cell wall
Source: eLife. 2024 Oct 1;13:RP99273. doi: 10.7554/eLife.99273 (PMC11444678; doi:10.7554/eLife.99273)

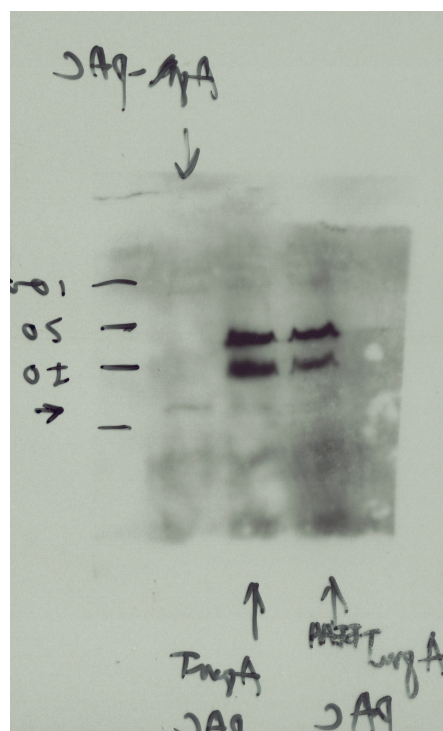

Supplement: Figure 5—source data 1. [file elife-99273-fig5-data1.pdf]

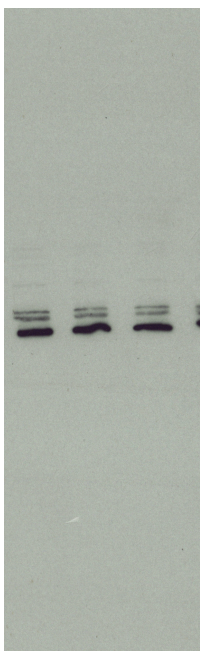

Supplement: Figure 5—source data 2. [file elife-99273-fig5-data2.pdf]

Scanned

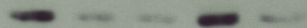

pellet Lysosome →

Supplement: Figure 5—source data 3. [file elife-99273-fig5-data3.pdf]

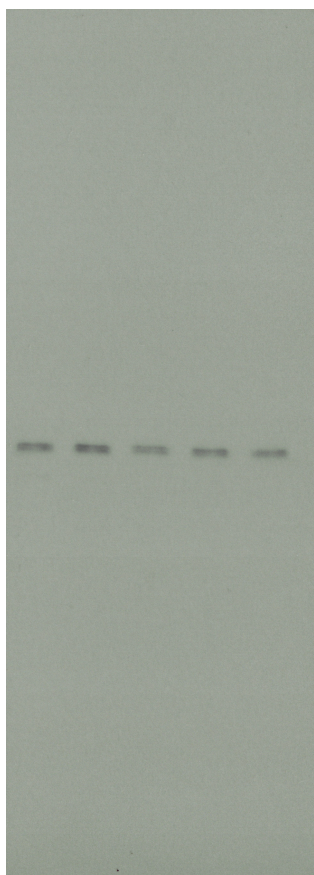

Supplement: Figure 5—source data 4. [file elife-99273-fig5-data4.pdf]

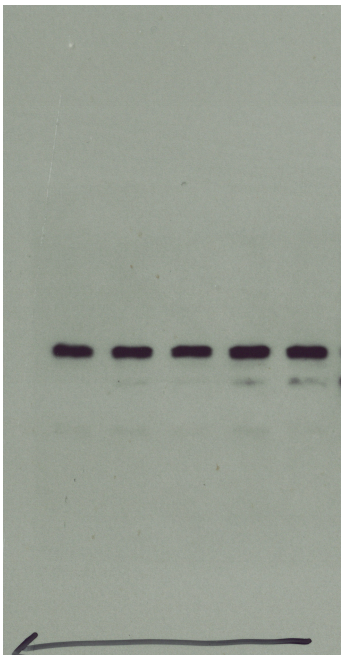

Supplement: Figure 5—source data 5. [file elife-99273-fig5-data5.pdf]
