# Supplementary material for: A lytic transglycosylase connects bacterial focal adhesion complexes to the peptidoglycan cell wall
Source: eLife. 2024 Oct 1;13:RP99273. doi: 10.7554/eLife.99273 (PMC11444678; doi:10.7554/eLife.99273)
Supplement: MDAR checklist [file elife-99273-mdarchecklist1.pdf]

Materials Design Analysis Reporting (MDAR)
Checklist for Authors

The MDAR framework establishes a minimum set of requirements in transparent reporting applicable to studies in the life sciences (see Statement of Task: doi:10.31222/osf.io/9sm4x.). The MDAR checklist is a tool for authors, editors, and others seeking to adopt the MDAR framework for transparent reporting in manuscripts and other outputs. Please refer to the MDAR Elaboration Document for additional context for the MDAR framework.

For all that apply, please note where in the manuscript the required information is provided.

Materials:

Newly created materials	indicate where provided: page no/section/legend)	n/a
The manuscript includes a dedicated "materials availability statement" providing transparent disclosure about availability of newly created materials including details on how materials can be accessed and describing any restrictions on access.	New stated in the first paragraph in the Mthods section, ”Newly created strains are available upon request to the corresponding author”.	

Antibodies	indicate where provided: page no/section/legend)	n/a
For commercial reagents, provide supplier name, catalogue number and RRID, if available.	We provided this information in the Key resources table.	

DNA and RNA sequences	indicate where provided: page no/section/legend)	n/a
**Short novel DNA or RNA including primers, probes:** Sequences should be included or deposited in a public repository.	N/A	

Cell materials	indicate where provided: page no/section/legend	n/a
**Cell lines:** Provide species information, strain. Provide accession number in repository **OR** supplier name, catalog number, clone number, **OR** RRID.	N/A	
**Primary cultures:** Provide species, strain, sex of origin, genetic modification status.	N/A	

Experimental animals	indicate where provided: page no/section/legend)	n/a
**Laboratory animals or Model organisms:** Provide species, strain, sex, age, genetic modification status. Provide accession number in repository **OR** supplier name, catalog number, clone number, **OR** RRID.	N/A	
**Animal observed in or captured from the field:** Provide species, sex, and age where possible.	N/A	

Plants and microbes	indicate where provided: page no/section/legend)	n/a
**Plants:** provide species and strain, ecotype and cultivar where relevant, unique accession number if available, and source (including location for collected wild specimens).	N/A	
**Microbes:** provide species and strain, unique accession number if available, and source.	Myxococcus xanthus strain DZ2	

Human research participants	indicate where provided: page no/section/legend) or state if these demographics were not collected	n/a
If collected and within the bounds of privacy constraints report on age, sex and gender or ethnicity for all study participants.	N/A	

Design:

Study protocol	indicate where provided: page no/section/legend)	n/a
If study protocol has been pre-registered, provide DOI. For clinical trials, provide the trial registration number **OR** cite DOI.	N/A	

Laboratory protocol	indicate where provided: page no/section/legend)	n/a
Provide DOI **OR** other citation details if detailed step-by-step protocols are available.	N/A	

Experimental study design (statistics details)		
**For in vivo studies:** State whether and how the following have been done	**indicate where provided: page no/section/legend. If it could have been done, but was not, write not done**	**n/a**
Sample size determination	No statistical methods were used to predetermine sample sizes for cell morphology measurements and single-particle tracking. Sample sizes were determined based on our expertise in bacterial cell biology, motility, and single-particle microscopy. For cell morphology measurements, motility, and single-particle tracking, data were collected from large numbers of cells (minimum 150 cells for morphology measurements, 40 cells for motility, and 100 cells for single-particle tracking). For each experiment, the results were confirmed by two additional biological replicates.	
Randomisation	As there is no allocation to subgroups, randomization is not applicable.	
Blinding	Two researchers ran analysis algorithms independently, one of them doing so in a blind fashion.	
Inclusion/exclusion criteria	No data were excluded.	

Sample definition and in-laboratory replication	indicate where provided: page no/section/legend	n/a
State number of times the experiment was replicated in laboratory.	Stated in each figure legend.	
Define whether data describe technical or biological replicates.	Stated in each figure legend.	

Ethics	indicate where provided: page no/section/legend	n/a
**Studies involving human participants:** State details of authority granting ethics approval (IRB or equivalent committee(s), provide reference number for approval.	N/A	
**Studies involving experimental animals:** State details of authority granting ethics approval (IRB or equivalent committee(s), provide reference number for approval.	N/A	
**Studies involving specimen and field samples:** State if relevant permits obtained, provide details of authority approving study; if none were required, explain why.	N/A	

Dual Use Research of Concern (DURC)	indicate where provided: page no/section/legend	n/a
If study is subject to dual use research of concern regulations, state the authority granting approval and reference number for the regulatory approval.	N/A	

Analysis:

Attrition	indicate where provided: page no/section/legend	n/a
Describe whether exclusion criteria were preestablished. Report if sample or data points were omitted from analysis. If yes report if this was due to attrition or intentional exclusion and provide justification.	No data were excluded.	

Statistics	indicate where provided: page no/section/legend	n/a
Describe statistical tests used and justify choice of tests.	Stated in each figure legend.	

Data availability	indicate where provided: page no/section/legend	n/a
For newly created and reused datasets, the manuscript includes a data availability statement that provides details for access or notes restrictions on access.	Indicated in the submission process on eLife website.	
If newly created datasets are publicly available, provide accession number in repository **OR** DOI **OR** URL and licensing details where available.	N/A	
If reused data is publicly available provide accession number in repository **OR** DOI **OR** URL, **OR** citation.	N/A	

Code availability	indicate where provided: page no/section/legend	n/a
For all newly generated custom computer code/software/mathematical algorithm or re-used code essential for replicating the main findings of the study, the manuscript includes a data availability statement that provides details for access or notes restrictions.	We reused published codes, which was stated in the last paragraph of the Methods section. The references were given.	
If newly generated code is publicly available, provide accession number in repository, **OR** DOI **OR** URL and licensing details where available. State any restrictions on code availability or accessibility.	N/A	
If reused code is publicly available provide accession number in repository **OR** DOI **OR** URL, **OR** citation.	DOI (https://doi.org/10.5281/zenodo.8234126) was given in ref 56.	

Reporting

MDAR framework recommends adoption of discipline-specific guidelines, established and endorsed through community initiatives. Journals have their own policy about requiring specific guidelines and recommendations to complement MDAR.

Adherence to community standards	indicate where provided: page no/section/legend	n/a
State if relevant guidelines (e.g., ICMJE, MIBBI, ARRIVE) have been followed, and whether a checklist (e.g., CONSORT, PRISMA, ARRIVE) is provided with the manuscript.	N/A	
